# Multidrug Resistence Prevalence in COVID Area

**DOI:** 10.3390/life11070601

**Published:** 2021-06-23

**Authors:** Caterina Aurilio, Pasquale Sansone, Antonella Paladini, Manlio Barbarisi, Francesco Coppolino, Vincenzo Pota, Maria Caterina Pace

**Affiliations:** 1 Department of Women, Child, General and Specialistic Surgery, University of Campania “L. Vanvitelli”, 80138 Napoli, Italy; pasquale.sansone@unicampania.it (P.S.); francesco.coppolino@unicampania.it (F.C.); vincenzo.pota@unicampania.it (V.P.); mariacaterina.pace@unicampania.it (M.C.P.); 2 Department of MESVA, University of L’Aquila, 67100 L’Aquila, Italy; antopaladini@gmail.com; 3 Department of Medical, Surgical, Neurological, Metabolic and Aging Sciences, University of Campania “Luigi Vanvitelli”, 80138 Naples, Italy; manlio.barbarisi@unicampania.it

**Keywords:** COVID-19, multidrug-resistant, risk factors, clinical characteristics

## Abstract

Coronavirus disease 2019 (COVID-19), caused by SARS-CoV-2, is often complicated by severe acute respiratory syndrome. The new coronavirus outbreak started in China in December 2019 and rapidly spread around the world. The high diffusibility of the virus was the reason for the outbreak of the pandemic viral disease, reaching more than 100 million infected people globally by the first three months of 2021. In the various treatments used up to now, the use of antimicrobial drugs for the management, especially of bacterial co-infections, is very frequent in patients admitted to intensive care. In addition, critically ill patients with SARS-CoV-2 infection are subjected to prolonged mechanical ventilation and other therapeutic procedures often responsible for developing hospital co-infections due to multidrug-resistant bacteria. Co-infections contribute to the increase in the morbidity–mortality of viral respiratory infections. We performed this study to review the recent articles published on the antibiotic bacterial resistance and viruses to predict risk factors of coronavirus disease 2019 and to assess the multidrug resistance in patients hospitalized in the COVID-19 area.

## 1. Introduction

Coronavirus disease 2019 (COVID-19), caused by severe acute respiratory syndrome coronavirus 2 (SARS-CoV-2), began in Wuhan, China in December 2019 and since then, has spread worldwide [1]. On 11 March 2020, the World Health Organization (WHO) declared the outbreak of a pandemic due to the SARS-CoV-2 infection [2].

COVID-19 is the most virulent infection of the last 100 years, with 107 million infected people and 237,000 deaths worldwide [2].

The virus enters cells by fusing with the cell membrane, releasing its genomic sequence into the cytoplasm [3]. SARS-CoV-2 penetrates the cells through the angiotensin-converting enzyme 2 (ACE2) protein, which is inactivated by the virus. The ACE2 protein has also the function of inactivating the angiotensin protein, which has a powerful anti-inflammatory effect. Since ACE2 is inactivated, it acts with severe inflammatory effects at the tissue levels. Once released into the cytoplasm, various elements are involved in viral replication and pathogenicity [3].

SARS-CoV-2 infection is responsible for acute respiratory failure, often requiring hospitalization in critical wards and respiratory support [4,5].

Several treatments have been proposed. Among these, the WHO authorized the use of the antiviral drug *remdesivir* and *dexamethasone* for the treatment of seriously ill or ventilated hospitalized patients [6,7,8]. Dexamethasone has been effective in several clinical trials reducing deaths by approximately 1/3 in severe ventilated patients with SARS-CoV-2 infections [8].

Several vaccines have been approved for emergency use by the Food and Drug Administration (FDA) and the European Medicines Agency (EMA). Pfizer–BioNTech [9,10], Moderna [11,12], Oxford–AstraZeneca [13] and Johnson & Johnson [14,15] COVID-19 vaccines are the main approved vaccines in USA and Europe. Many others are authorized by at least one national regulatory authority for public use. In addition, the adoption of preventive measures, especially hygiene procedures such as healthcare workers’ hand washing and protective equipment use, may reduce nosocomial microorganism diffusion and the spread of the infection [16,17].

In addition, critically ill patients with SARS-CoV-2 infection are subjected to prolonged mechanical ventilation and peripheral and central venous access, often responsible for developing hospital co-infections due to multidrug-resistant bacteria (MDRB).

Historically, multidrug resistance represents a major health crisis and requires the development of new antibiotics. Antibiotic bacterial resistance has become an urgent problem for environmental safety [18], as bacteria are modifiable organisms that can become resistant to novel therapeutic agents [19]. Penicillin was discovered in 1928 and after 20 years, an enzyme (beta lactamase) that inactivates it; hence, resistance development against penicillin was discovered [20]. Fluoroquinolones have been widely used for several years in various kinds of infections, and resistance has been evidenced as well [21,22].

Aims. The aim of this study is to define the antibiotics use in patients hospitalized in wards or in intensive care units (ICUs) for SARS-CoV2 infection and to evidence the possibility of reducing co-infections and multidrug bacterial resistance (MDBR).

## 2. Co-Infections in COVID-19 Patients

In recent years and especially during the COVID-19 pandemic, the search for new antimicrobial agents for the treatment of multi-resistant microorganisms has become crucial. Bacterial infection and superinfection, an additional infection that happens during or immediately after an existing infection, contribute to the increased morbidity–mortality of viral respiratory infections [23]. This occurs for many patients with SARS-CoV-2 infection, which worsens the course of the disease [24].

Feng Y. et al. compared the clinical features and treatments on 476 patients with SARS-CoV-2 infection recruited from three different cities in China in the first months of 2020 [25]. The patients were divided into three groups according to the intensity of pathology: critical (first group), severe (second group) and moderate (third group). Multiple organ dysfunctions and the involvement of multiple lung lobes were associated with higher critically disease.

The patients with critical disease had the highest percentage of bacterial superinfections (34.5%) compared to the moderately (3.9%) and severely (8.3%) ill patient groups. Most of the patients (67.0%) received antibacterial therapy and steroids in the first and second group; more patients received antiviral agents in the third group than in the second group. Patients of the second and third group treated with antimicrobial compounds or corticosteroids were hospitalized less than others who had not received them. Adults over 75 years old with COVID-19 had poor outcome and the in-hospital mortality rate among critical patients was 41.1% [25].

In another retrospective cohort study, 191 patients with laboratory-confirmed COVID-19 in two hospitals in Wuhan, China were analyzed by Zhou F. et al. to explore the risk factors for mortality of adult inpatients [26]. A total of 181 patients (95%) were treated with antimicrobial agents and 41 patients (21%) with antiviral agents. Although bacterial infections are a principal cause of sepsis, viral infection can induce sepsis as well [27]. Sepsis occurrs in nearly 40% of adults with community-acquired pneumonia (CAP) due to viral infection. In this study, more than 50% of patients developed sepsis or a higher Sequential Organ Failure Assessment (SOFA) score on hospitalization, with a high percentage of deaths among patients.

Patients with SARS-CoV-2 infection and severe illness are treated for a long time with mechanical ventilation, often responsible for further hospital infection, and then SARS-CoV-2 patients are often treated in ICUs for bacterial infections with high doses of antibiotics [27,28,29].

During the New York City pandemic surge (USA) from April–July 2020, bacterial and fungal infections were reported in COVID-19 patients due to multidrug-resistant (MDR) Gram-negative bacteria (GNB) in two different ICUs [30].

Critical illness requiring extracorporeal membrane oxygenation (ECMO) support or renal replacement therapy (RRT), and high antibiotic use were associated with MDR infections [31]. In the same study, Patel A et al. screened a total of 71 patients that had positive cultures for resistant GNB, involving 44 *Escherichia coli*, 27 MDR *Pseudomonas aeruginosa*, and 27 MDR *Acinetobacter baumanii*. Twenty-four patients (34%) were superinfected with >1 resistant GNB. Of the 71 patients, 69 (97%) had received antibiotics before the first positive resistant GNB culture; 23 (32%) patients in-hospitalized died [31].

In a recent report, Ramadan HK et al. included 260 patients with SARS-CoV-2 in order to predict multidrug resistance in patients admitted to ICUs in Upper Egypt [32]. A total of 51.5% of these patients developed moderate illness, 25.4% mild and 23% severe/critical illness; 28 patients (10.7%) developed bacterial and/or fungal infections. The results of the bacteriological investigations highlighted 42 positive results, of which 37 were bacterial microorganisms and 5 fungal microorganisms. In the microbiological cultures, Gram-negative bacteria were more numerous 30/42 (71.4%) than Gram-positive bacteria. All the Gram-positive bacteria were 100% resistant to amoxicillin, gentamicin and piperacillin/tazobactam. On the other hand, the Gram-negative bacteria were mostly extended spectrum beta-lactamases (ESBL) and carbapenase producers. Different resistance-associated genes were evaluated, and among these New Delhi Metallo–beta-lactamase (NDM-1) was the most predominant gene (16/29) isolate.

Bacterial infection associated with a viral infection worsens the clinical disease and enhances mortality in patients hospitalized in ICUs [33]. Most of the co-infections in different studies were observed from first to seventh day of SARS-CoV-2 starting and impacted on the degree of disease and mortality. Very often in the course of an infection, viral respiratory pathogens join with bacterial agents and so they reduce their mucociliary clearance [34]. To date, no new drugs against Gram-negative resistant bacteria have been produced, and so we observe a prevalence of them. Several genes resistant to antimicrobial agents have been markedly discovered in a number of patients, and mostly Gram-negative resistant bacteria were ESBL and/or carbapenemase producers.

The presence of both ESBL and MBL enzymes is the basis of antibiotic resistance, and carbapenemes are also part of these. This is one of the main reasons for the failure of an antibiotic therapy [35]. For patients with COVID-19 disease, who show coinfections with MDR pathogens, the antimicrobial sensitive report is mandatory, as well as the subsequent administration of antibiotics, as infection control strategies [36,37].

Bentivegna et al. developed a retrospective study to establish whether the incidence of MDR bacteria were lower due to pandemic-related preventive measures compared to previous years. In the pre-pandemic years, they monitored hospital discharges of 1617 patients over a period of 4 months (March–June) from 2017 to 2020 [38]. In their hospital, only basic prevention measures were adopted; instead, during the pandemic, several additional measures were used, according to the WHO [39,40].

The incidence of MDBR was evaluated in 2020 compared to the years 2017–2019: the incidence of total infection by MDR bacteria was 45.2 cases per 100 discharges during 2017, 44.2 during 2018, 41.4 during 2019, 19.2 during 2020 in non-COVID-19 wards and 29.3 during 2020 in COVID-19 wards. The extended-spectrum beta-lactamase Klebsiella pneumoniae showed the highest incidence.

MDR bacterial infections were significantly reduced in the year 2020 compared to the years 2017–2019. The same reduction was reported in a study by Wee et al. [41]. Viral or bacterial contact transmission can occur after touching one’s mask or with poor hand hygiene and constitutes an increasing source of hospital-contaminated biological waste. During the pandemic, proper hygiene procedures and protective measures may decrease the spread of the infection. Furthermore, patients with SARS-CoV-2 infection have a higher incidence of MDR bacterial infections matched with non-COVID-19 patients of the same year. Staphylococcus Aureus, extended-spectrum beta-lactamase Klebsiella pneumoniae, Clostridium difficile and Acinetobacter baumanii were the MDBR that were isolated. This result could be explained by the intensive use of broad-spectrum antibiotic agents in patients with SARS-CoV-2 infection since the first day of admission in ICUs [41].

Many authors believe that, in patients with SARS-CoV-2 infection, especially in the first months of pandemic, the treatment with azithromycin, which acts primarily against Gram-positive-bacteria, may be the cause of the predominance of Gram-negative coinfections. Moreover, a compromised immune system in patients with severe forms of SARS-CoV-2 infection represents an significant risk factor for MDR bacterial infections [42,43].

In a similar study, the presence of MDR bacteria was reported in critically ill people with acquired SARS-CoV-2 admitted in ICUs [44]. Bogossian et al. concluded that the acquisition rate of MDR bacteria in ICU patients during the COVID-19 pandemic was not different from the pre-pandemic period.

In another study, the authors evaluated the percentage of MDR bacteria in patients hospitalized in ICUs for SARS-CoV-2 infection and the outcome for a long time of mechanical ventilation [45], with patients admitted in the same critical ward for cerebral hemorrhage (SAH) [46] scheduled in a preexisting SAH institutional database cohort matched for the same criteria [47,48]. Among all the people hospitalized in ICUs for SARS-CoV-2 infection, 33% were infected with 31 MDR bacteria while they were hospitalized.

In Table 1, we report an overview of the main articles reporting the prevalence of MDR in the COVID area.

Isolated MDR bacteria were mostly AmpC enterobacteriaceae (39%) and beta-lactamase enterobacteriaceae (29%) [36,49].

The authors report a high rate of MDR bacteria in patients with SARS CoV-2, which is not statistically different from the control group. A large antibiotic use in patients with initial COVID-19 symptoms, even where there is bacterial infection, significantly increases the presence of MDR bacteria [45].

The pre-emptive treatment with antimicrobial agents in patients with SARS-CoV-2 infection and the possible impact on levels of resistance has been evaluated in a recent commentary [50] A high number of worldwide patients with SARS-CoV-2 infection hospitalized in wards or in ICUs are at serious risk of co-infections. Lansbury et al., in a systematic review on 2834 patients, observed a secondary infection in 7% of hospitalized patients, and in 14% of those in wards and in ICUs [43]. Lansbury et al. are in agreement with the review of Langford et al. on bacterial co-infection at admission and secondary infection in patients with SARS-CoV-2 [51].

Recently, the considerable use of antimicrobial compounds in patients with SARS-CoV-2 infection in the first phase of the pandemic may influence the onset of bacterial resistance [52,53]. Many countries have reported an antibiotic resistance, and the data they provide show an exponential number of bacterial infections. Additionally, the WHO Director-General, Dr Tedros Adhanom Gebreyesus said “*As we gather more evidence, we see more clearly and more worryingly how fast we are losing critically important antimicrobial medicines all over the world*”.

WHO is worried about the abuse of antibiotics during the SARS-CoV-2 pandemic. A wide quantity of reviews report that only a limited number of these patients need antibiotics to heal bacterial infections; therefore, WHO published guidelines not to treat patients with mild infection with antimicrobial compounds and similarly, patients with moderate illness, except on clinical indication [54]. Antimicrobial agents have to be wisely used, while empirical therapy should be rarely reserved to those cases which have a high chance of bacterial infection. Hence, it is mandatory to re-evaluate empirical antibiotics management according to microbiological results [55].

## 3. Conclusions

Bacterial or fungal co-infection for patients presenting coronavirus infections appears to be low. The presence of the MDRB was numerous for people hospitalized in intensive care with SARS-CoV-2. However, long hospitalized and ventilated patients did not show statistical difference to those admitted in previous pre-pandemic years in an ICU.

Maintaining a high level of healthcare workers’ hand hygiene and preventive hygiene procedures may reduce the spread of hospital-associated infections. However, to allow a statistical decrease in MDBR, it is necessary to increase antimicrobial stewardship programs, designed to promote the right use of antimicrobial compounds and a better strategy to face antibiotic resistance in hospitalized COVID-19 patients.

The rising number of MDRB and our decreasing capacity to eradicate them have notable influence on outcome of patients suffering from SARS-CoV 2 disease that are more exposed bacterial infections.

As COVID-19 proceeds to implement this important problem, we quickly need to investigate the effects of bacterial co-infections during viral infections and to research new antibiotics to eradicate multidrug-resistant pathogens. Furthermore, with preventive measures, it is possible to challenge the spread of MDRB.

## Figures and Tables

**Table 1:** Overview of the main articles reporting the prevalence of MDR in the COVID area.

Author, Year	Country	Type of Publication	No. Coinfections	No. MDR Coinfections	Microorganism (No.)	Death (No.)
Bogossian EG et al., 2020	Belgium	Case control study	72	31	MDR Gram-negative (?): MDR *Enterobacteriaceae* (?)	25
Nori P. et al., 2021	USA	Retrospective observational study	152	24	MDR Gram-negative (24): carbapenem-resistant Enterobacteriaceae (*10*)	15
Ramadan HK et al., 2020	Egypt	Case series	28	?	MDR Gram-negative (?)	N/D
Patel A et al., 2021	USA	Retrospective observational study	87	87	MDR Gram-negative (?): MDR E. Coli (33), cefepime-resistant E. Coli (11), MDR *P. aeruginosa* (*27*), MDR *A. baumannii* (27)	N/D
Zhu X et al., 2020	China	Retrospective observational study	243	N/D	N/D	None

N/D, no data.

## Data Availability

Not applicable.
